# Enhanced Photocatalytic Degradation of Malachite Green Dye Using Silver–Manganese Oxide Nanoparticles

**DOI:** 10.3390/molecules28176241

**Published:** 2023-08-25

**Authors:** Zhong Xu, Noor Zada, Fazal Habib, Hamid Ullah, Kashif Hussain, Naveed Ullah, Marwa Bibi, Maria Bibi, Huma Ghani, Suliman Khan, Khitab Hussain, Xinyan Cai, Habib Ullah

**Affiliations:** 1 Qingdao University of Science and Technology, Qingdao 266001, China; 2 Department of Chemistry, Government Post Graduate College, Lower Dir, Timergara 18300, Pakistanhameedullah@pgct.edu.pk (H.U.); bibimarwa@pgct.edu.pk (M.B.); bibimaria@pgct.edu.pk (M.B.);; 3 Shandong Institute of Scientific and Technical Information, Jinan 250000, China; 4 College of Environmental and Resource Science, Zhejiang University, Hangzhou 310058, China; habib901@zju.edu.cn

**Keywords:** malachite green, photocatalytic degradation, silver–manganese oxide nanoparticles, wastewater remediation

## Abstract

Efficient and excellent nanoparticles are required for the degradation of organic dyes in photocatalysis. In this study, silver–manganese oxide nanoparticles (Ag-Mn-NPs) were synthesized through a wet chemical precipitation method and characterized as an advanced catalyst that has enhanced photocatalytic activity under sunlight irradiation. The nanoparticles were characterized using scanning electron microscopy (SEM), XRD, UV–vis light spectra, and energy-dispersive X-ray (EDX) spectroscopy, revealing their spherical and agglomerated form. The EDX spectra confirmed the composition of the nanoparticles, indicating their presence in oxide form. These bimetallic oxide nanoparticles were employed as photocatalysts for the degradation of malachite green (MG) dye under sunlight irradiation in an aqueous medium. The study investigated the effects of various parameters, such as irradiation time, catalyst dosage, recovered catalyst dosage, dye concentration, and pH, on the dye’s photodegradation. The results showed that Ag-Mn oxide nanoparticles exhibited high photocatalytic activity, degrading 92% of the dye in 100 min. A longer irradiation time led to increased dye degradation. Moreover, a higher catalyst dosage resulted in a higher dye degradation percentage, with 91% degradation achieved using 0.0017 g of the photocatalyst in 60 min. Increasing the pH of the medium also enhanced the dye degradation, with 99% degradation achieved at pH 10 in 60 min. However, the photodegradation rate decreased with increasing dye concentration. The Ag-Mn oxide nanoparticles demonstrate excellent potential as a reliable visible-light-responsive photocatalyst for the efficient degradation of organic pollutants in wastewater treatment.

## 1. Introduction

In the wake of global population growth and burgeoning industrial activities, the contamination of water bodies with diverse pollutants has risen to alarming levels. This rampant pollution poses a significant threat to water resources by exacerbating water scarcity and jeopardizing water quality. The urgency to combat this problem is evident, given its direct repercussions on water-supply security, human health, natural ecosystems, and overall quality of life. Given that water is the source of life itself, tackling the issue of severe water pollution is of utmost importance. The quest for clean water is pivotal for not only meeting essential human needs but also upholding the wellbeing of other living organisms and sustaining various commercial operations [1,2]. The escalating issue of water pollution, primarily driven by the presence of non-biodegradable pollutants from diverse industries, has become a pressing concern [3]. Among the most prevalent contaminants, organic dyes pose a significant threat. These dyes not only disrupt the photosynthesis process but also have adverse effects on human health, such as skin irritation, allergies, and even cancer [4]. Approximately 15% of the dyes released into water bodies through textile effluents during the dyeing process contribute to this pollution. To solve this problem, various approaches, including physicochemical and biological processes, have been developed for dye treatment, each with its own advantages and disadvantages [5].

In previous studies, efforts directed toward addressing dye degradation in contaminated wastewater have been noteworthy, encompassing chemical and physical remediation techniques, including flocculation, electrocoagulation, activated-carbon sorption, and UV–visible degradation. However, each of these methods possesses certain drawbacks that hinder their widespread adoption [6,7,8]. These limitations often include challenges in operational control, incomplete degradation, and high operational costs. Additionally, some of these methods might not be environmentally sustainable owing to the generation of secondary pollutants. The emergence of nanotechnology has opened new possibilities for wastewater treatment, with silver nanoparticles proving to be effective in this regard. Nanomaterials offer unique advantages owing to their specific characteristics, such as larger surface area and smaller size, distribution, and morphology. These intrinsic properties make nanoparticles highly suitable for catalytic degradation applications [9].

Photocatalytic degradation is a widely recognized and effective technique for completely breaking down organic compounds present in contaminated wastewater. By employing an appropriate photocatalyst and harnessing light radiation, this process generates highly reactive hydroxyl (OH•) radicals. These radicals can transform water pollutants into relatively benign end products, such as CO_2_, H_2_O, and other inorganic ions [10,11]. This conversion ensures the safety of both human beings and the environment. Photocatalytic degradation offers several advantages compared to other conventional methods. It is an effective and candid instrumental technique, with easy-to-control operations and nonselective oxidation. Additionally, it is cost-effective and capable of achieving complete mineralization and degradation of synthetic organic dyes [12]. This process relies on the presence of a semiconductor photocatalyst that becomes activated by absorbing photons. Remarkably, the photocatalyst can accelerate the reaction without being consumed [13]. Metal nanoparticles are the most used photocatalysts, and their properties directly depend on factors such as particle shape, size, geometry, and morphology [14]. Nanoparticles are tiny grains with sizes below 100 nm and have attracted significant attention owing to their unique chemical and physical properties. They have demonstrated potential applications in various fields, including medicine, solar cells, and nanodevices [15].

Malachite green (MG) is a valuable dye that stands out for its affordability, wide availability, and effectiveness. It serves multiple purposes, functioning as a food-coloring agent, food additive, medical disinfectant, and anthelminthic agent. Additionally, it finds application in various industries, such as silk, wool, jute, leather, cotton, paper, and acrylic, where it is used for dyeing processes [16]. In the global agricultural industry, malachite green is extensively utilized as a biocide, proving highly effective against significant protozoal and fungal infections. Notably, it is employed to control skin flukes and gill flukes, providing effective management of these conditions [17].

The current research seeks to overcome these drawbacks by employing an innovative approach. By synthesizing silver–manganese bimetallic oxide nanoparticles (Ag-Mn-NPs) as a photocatalyst through a wet chemical precipitation method, this study aims to enhance the degradation efficiency of organic dyes. The primary advantage of such catalysts lies in leveraging the synergistic effects of bimetallic catalysts, where the high bandgap of one catalyst (e.g., manganese oxide) is offset by the lower bandgap of the other (e.g., silver nanoparticles). This leads to improved electron excitation, thereby enhancing the catalytic activity of the mixed bimetallic nanoparticles. This approach addresses the limitations posed by single-component catalysts and promises enhanced photocatalytic performance. Furthermore, the meticulous examination of the morphology, structure, and elemental composition of the photocatalyst using scanning electron microscopy (SEM), XRD, UV–visible spectra, and energy-dispersive X-ray spectroscopy ensures a comprehensive understanding of the catalyst’s characteristics. By focusing on these aspects, the study aims to optimize the catalyst design and composition to maximize efficiency. The objectives of this study are multifaceted. First, it aims to develop a cost-effective and efficient photocatalyst for the degradation of malachite green dye. By focusing on the degradation of a widely used dye with multiple applications, the study addresses a real-world pollutant challenge. Second, the research delves into parameters that influence degradation efficiency, including time, medium pH, photocatalyst dosage, amount of recovered photocatalyst, and dye concentration. By systematically exploring these factors, the study aims to provide insights into optimal conditions for dye degradation, thus advancing our ability to combat water pollution.

In summary, the current research is a significant step forward from previous studies by introducing bimetallic oxide nanoparticles as an innovative approach to enhance photocatalytic degradation. The characterization of the synthesized nanoparticles and the systematic exploration of influential factors contribute to a more holistic understanding of the process. By addressing drawbacks observed in earlier methods and seeking enhanced efficiency, this study stands to contribute meaningfully to the field of water pollution mitigation and sustainable water resource management.

## 2. Results and Discussion

### 2.1. Scanning Electron Microscopy (SEM) Analysis of the Ag-Mn Oxide Nanoparticles

**Figure 1:**
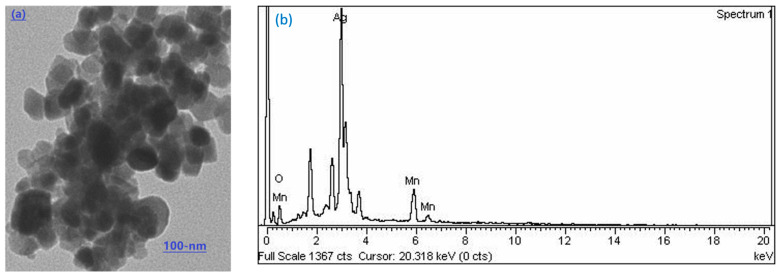
Scanning electron microscopy image of Ag-Mn oxide (**a**) and EDX spectrum (**b**).

**Figure 2:**
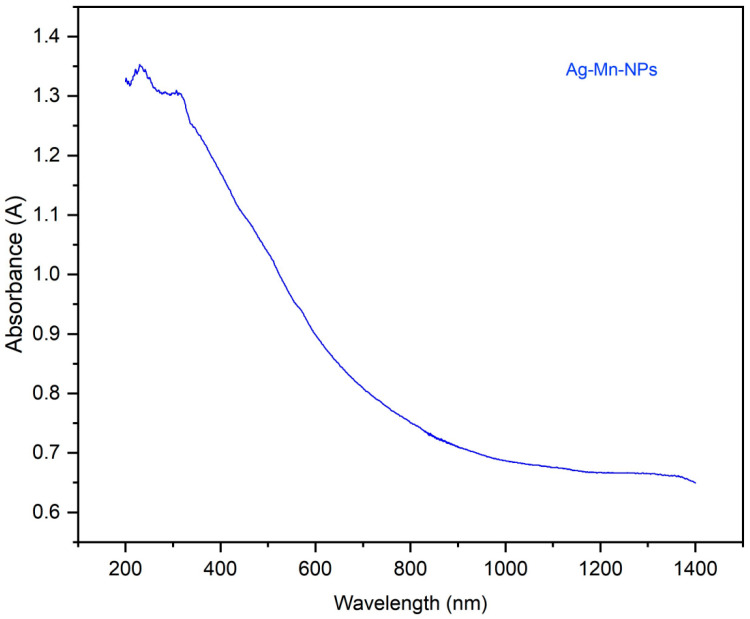
UV–visible graph for the prepared photocatalyst (Ag-Mn bimetallic nanoparticles).

**Figure 3:**
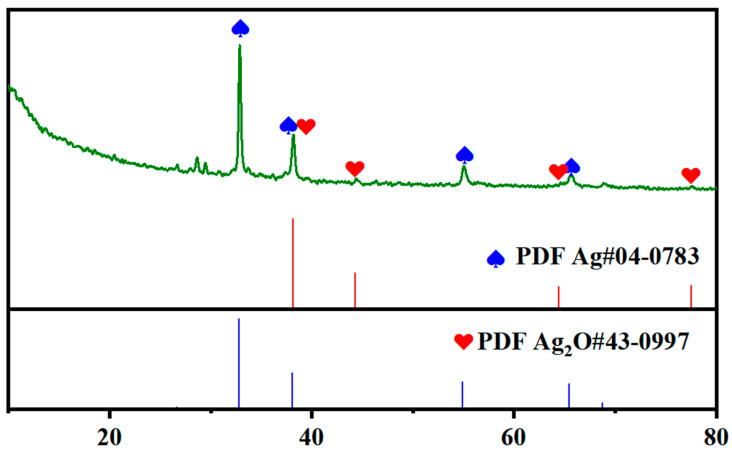
XRD analysis of the prepared photocatalyst.

The surface morphology of the Ag-Mn oxide nanoparticles was examined using SEM (Scanning Electron Microscopy). Figure 1a shows the particle grains of Ag-Mn oxide nanoparticles at 100 nm, demonstrating the successful synthesis of the nanostructures, with a particle size in the nanometer range. The elemental silver and manganese in the prepared samples were confirmed using EDX analysis, and the corresponding spectra for Ag-Mn oxide nanoparticles are presented in Figure 1b. The EDX spectrum displays prominent peaks for silver (Ag) and oxygen, while shorter peaks indicate the presence of manganese. A significant peak at approximately 3 keV was observed in the EDX profile of the Ag-Mn oxide nanoparticles, confirming the presence of elemental silver in the synthesized nanoparticles [18]. The presence of oxygen confirms that both the Ag and Mn nanoparticles are in the oxide form. Figure 2 displays the UV–visible graph showing the optical properties of the prepared photocatalyst, which consists of Ag-Mn bimetallic nanoparticles. This graph provides insights into how the material interacts with ultraviolet (UV) and visible light, offering information about its absorbance and reflection characteristics across a range of wavelengths. UV–visible spectroscopy is commonly used to study the electronic transitions and surface plasmon resonances of nanoparticles, helping to understand their potential for applications in various fields, such as catalysis, sensing, and energy conversion. Moreover, the crystallinity of the synthesized silver–manganese bimetallic nanoparticles was verified using X-ray diffraction (XRD) analysis. The XRD profile exhibited four distinct peaks (two long and two moderately long) that are characteristic of the prepared nanoparticles (as shown in Figure 3). These peaks precisely matched the standard values for bimetallic nanoparticles.

### 2.2. Photocatalytic Degradation of Malachite Green Dye

The discharge of dye effluents from the textile industry poses a significant threat to water pollution. Various industries release dyes and other organic compounds as waste products, which have detrimental effects on humans, animals, and plants. Malachite green, being a stable and basic dye with aromatic compounds, is particularly problematic. Owing to its stability and resistance to heat and light, the treatment of malachite green dye (MGD) is highly necessary. Silver nanoparticles (AgNPs) have emerged as effective photocatalysts for treating various dye pollutants. They offer several advantages, such as a high surface area-to-volume ratio, nontoxicity, cost-effectiveness, and a novel approach for addressing dye pollution. The utilization of AgNPs as photocatalysts provides an efficient and environmentally friendly method for treating dye contaminants [19]. In the present work, the photocatalytic activity of the synthesized Ag-Mn oxide nanoparticles was investigated utilizing them to catalytically degrade malachite green dye in an aqueous medium under sunlight irradiation.

#### 2.2.1. Effect of Time on the Photocatalytic Degradation

**Figure 4:**
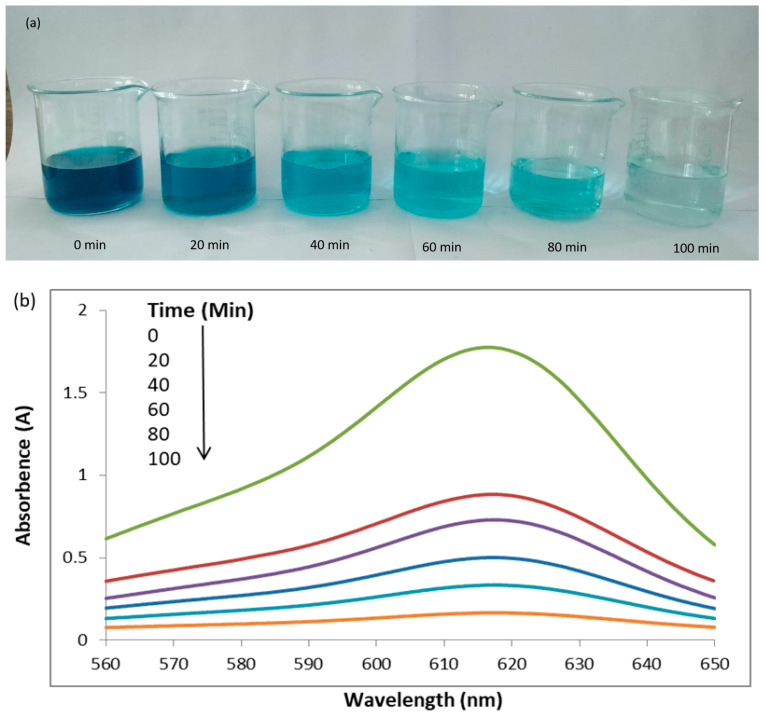

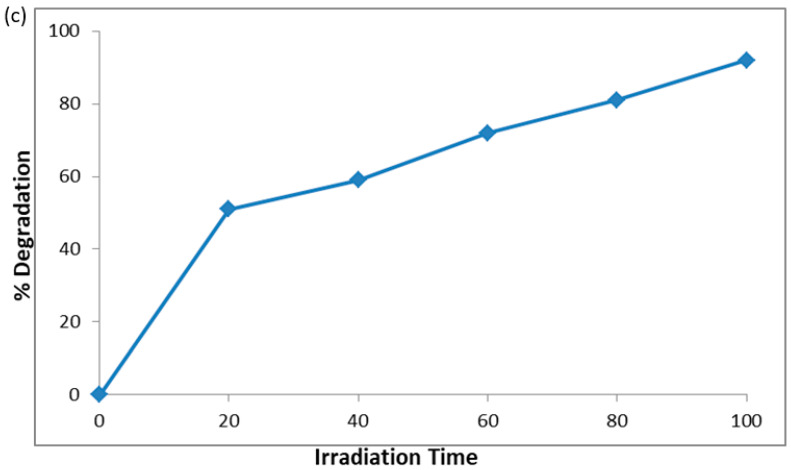
Photographic images of dilution and catalytic behavior of malachite green (MG) after degradation at different time intervals (**a**) UV–visible graph of MG dye degradation at different time intervals (**b**) and percentage degradation of MG dye at different time intervals (**c**).

Aqueous solutions containing 25 ppm of MG dye were subjected to degradation by Ag-Mn oxide nanoparticles for different durations. Visual representations captured through photographic images depict the dilution and catalytic behavior of MG during its degradation process. These images show the evolution of MG’s appearance at different time intervals: 0, 20, 40, 60, 80, and 100 min (Figure 4a). Initially, at the start of the degradation process (0 min), the photographic image exhibits a deep and intense green that is characteristic of the unaltered MG dye. As the degradation process unfolds, an observable transformation occurs. At subsequent time intervals, spanning 20, 40, 60, 80, and 100 min, a significant change in the color of the dye becomes apparent. As time progresses, the color of the MG dye gradually shifts from a darker, more saturated hue toward a progressively lighter shade. This shift from dark to light coloration signifies the ongoing breakdown of dye molecules because of catalytic activity. The transition from an initially concentrated and intense color to a more subdued and lighter tone indicates the effective degradation of MG into simpler molecular components.

The UV–visible graph shows a gradual increase in the photodegradation of the malachite green dye at longer irradiation times. The degradation of MG was evaluated based on the relative intensity of its UV–vis spectral peak, which exhibited the maximum absorbance at 617 nm (Figure 4b). Figure 4c illustrates the percentage degradation of the MG dye. The graph clearly shows that the percentage of degradation increases as the sunlight irradiation time increases. Specifically, the dye exhibited degradation rates of 51%, 59%, 72%, 81.5%, and 92% at 20, 40, 60, 80, and 100 min, respectively. In essence, this UV–visible graph serves as visual proof of the photocatalytic degradation process’s effectiveness. The gradual increase in the degradation percentage at longer irradiation times confirms the sustained and consistent breakdown of MG dye molecules. This graph offers a quantitative representation of the degradation kinetics, reflecting the relationship between the sunlight exposure duration and dye degradation efficiency.

#### 2.2.2. Effect of Dye Concentration on Photocatalytic Degradation

**Figure 5:**
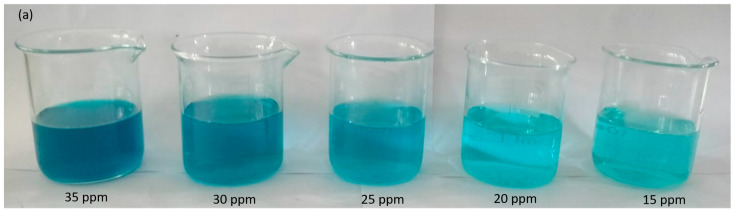

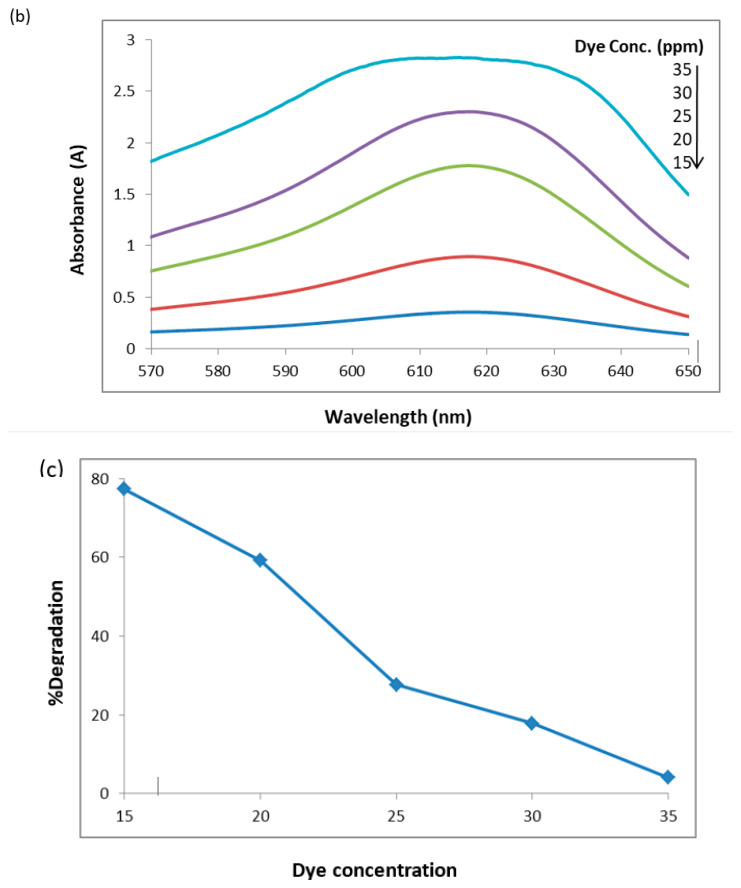
Photographic images of dilutions at different dye concentrations (**a**) UV–visible graph for 15, 20, 25, 30, and 35 ppm solutions of MG dye degraded for 60 min (**b**) and percentage degradation of malachite green dye at 60 min (**c**).

To assess the impact of the MG dye concentration on the photocatalytic degradation process, the initial dye concentration was varied within the range 15–35 ppm. The study aimed to determine a suitable dye concentration while maintaining a constant amount of catalyst (0.0003 g) for a duration of 60 min. The photographic images capture the dilution phenomenon occurring within solutions of MG dye at varying concentrations: 15, 20, 25, 30, and 35 ppm, as depicted in Figure 5a. These images provide a visual representation of the dye’s behavior after undergoing degradation for a fixed duration of 60 min. Starting with the 15-ppm dye solution, the photographic image displays the color and intensity of the dye in its original state. As the dye concentration increases incrementally to 20 ppm, 25 ppm, 30 ppm, and 35 ppm, each subsequent image portrays a gradually deepening and more concentrated coloration. The degradation process, which takes place over the course of 60 min, induces changes in the appearance of the dye solutions. The photographic images capture this alteration, show the progressive reduction in the dye’s vibrancy and color intensity across the range of concentrations.

Figure 5b shows that the degradation rate decreased by increasing the dye concentration. The UV–visible graph shows that the photodegradation of the MG dye decreased by increasing the dye concentration. According to the Beer–Lambert law, the high dye concentration decreases the path length of the entering photons, which results in lower photon adsorption on the catalyst surface and, consequently, low photodegradation occurs [20]. The reduction in degradation of the MG dye with increasing dye concentration can be attributed to a higher availability of dye molecules for excitation and energy transfer at higher initial dye concentrations. This phenomenon may be linked to the formation of multiple monolayers of adsorbed dye on the nanoparticles, which is more favorable at higher dye concentrations. Until the critical level is reached, the surface is not completely covered, resulting in constant reaction rates [21]. However, beyond this critical level, further increases in the initial dye concentration lead to a significant decline in degradation efficiency for several reasons. As the dye concentration increases, more dye molecules are adsorbed on the photocatalytic material surface, resulting in a greater absorption of UV light by the dye molecules rather than the nanoparticles themselves. Consequently, there is a decrease in the penetration of light at the photocatalytic surface, leading to a reduction in the generation of hydroxyl radicals and, subsequently, a decline in dye degradation efficiency. Additionally, the adsorbed dye on the photocatalyst surface hinders the reaction between adsorbed molecules with photoinduced positive holes or hydroxyl radicals. Hence, as the initial dye concentration increases, a larger catalyst surface area is required to provide sufficient hydroxyl radicals for effectively attacking the available dye molecules and facilitating complete dye degradation [22].

Figure 5c shows the percentage degradation of the MG dye, which decreases as the dye concentration increases. The percentage degradation at 60 min of 15, 20, 25, 30, and 35 ppm dye solutions is 77%, 59%, 27.6%, 17.8%, and 4% respectively.

#### 2.2.3. Effect of pH Solution on Photodegradation

**Figure 6:**
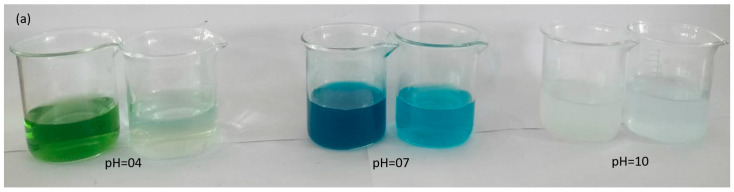

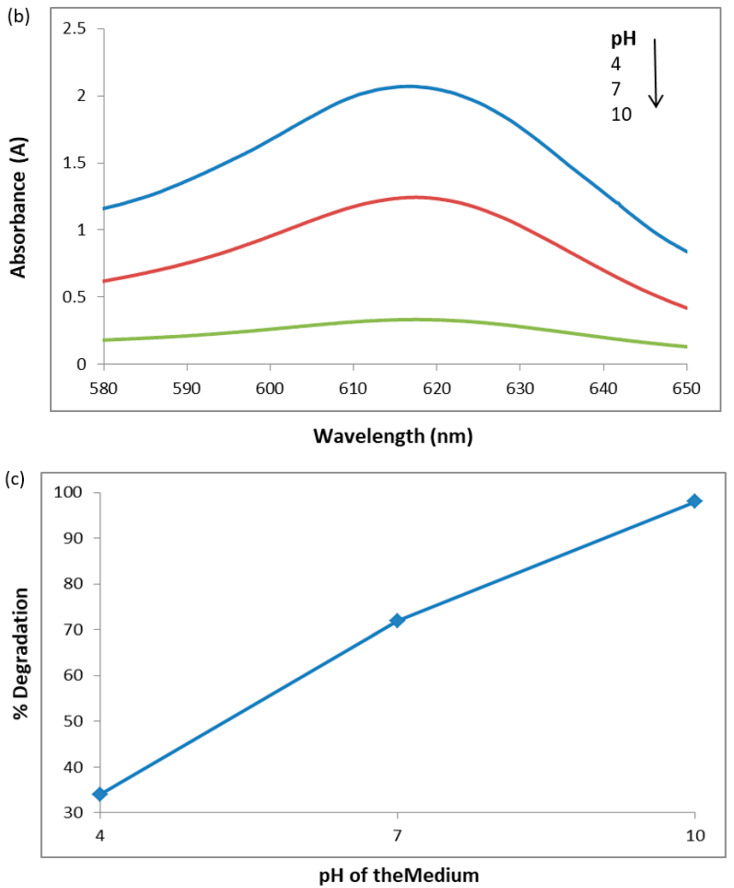
Original solutions before degradation and after degradation at pH 4, 7, and 10. Left side of each image pair indicates original solutions before degradation, while the right side represents original solutions after degradation (**a**) UV–visible graph of malachite green dye degraded for 60 min at different pH levels of the medium (**b**) and percentage degradation of malachite green dye for 60 min at different pH levels (**c**).

The pH of the chemical reaction system plays a critical role in determining the surface properties of the solid catalyst and the characteristics of the dissolved ions. It exerts significant influence, making it a crucial factor to consider [23]. The degradation of dye is influenced by the pH of the solution, as effluents from various industries, such as dyes, paints, and textiles, are released at different pH levels. Therefore, the impact of pH on the degradation rate of the dye was examined at pH 4, 7, and 10. The pH level plays a crucial role in the photodegradation process of textile waste and the generation of hydroxyl radicals. The comparative photographic images capture the transformation of the original solutions before and after degradation at different pH levels: 4, 7, and 10 (Figure 6a). The arrangement of the images highlights the distinct changes in color and appearance between the predegradation and postdegradation states. On the left side of each paired image, the original solutions are depicted before the degradation process. These solutions are characterized by their initial dark and intense coloration, which represents the unaltered state of the dye at the respective pH levels. The darkness of the color indicates the concentration and vibrancy of the dye molecules present in the solution. Conversely, on the right side of each image pair, the solutions are shown after degradation at the specified pH levels. In these images, the color of the solutions appears notably lighter and less intense compared to their original state. This change in color is a direct result of the photocatalytic degradation process that the dye molecules have undergone.

In Figure 6b, the UV–visible graph indicates the relationship between the photodegradation rate of the MG dye and the pH of the medium. The graph indicates that as the pH of the medium increases, the photocatalytic degradation of the dye also increases. This can be attributed to the higher formation of active •OH radicals at higher pH levels, leading to an enhanced degradation rate. Figure 6c presents the percentage degradation of the malachite green dye at pH 4, 7, and 10. The graph clearly shows that the percentage degradation of the dye increases with an increase in pH. After 60 min of degradation, the percentage degradations at pH 4, 7, and 10 are 34%, 72%, and 99% respectively.

#### 2.2.4. Effect of Catalyst Dosage on Photodegradation

**Figure 7:**
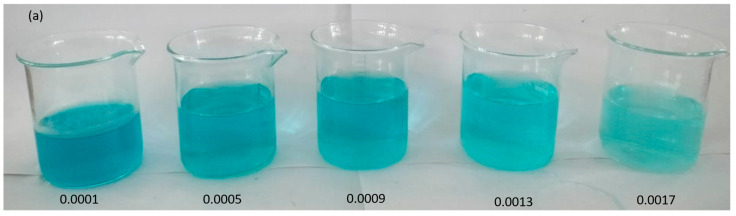

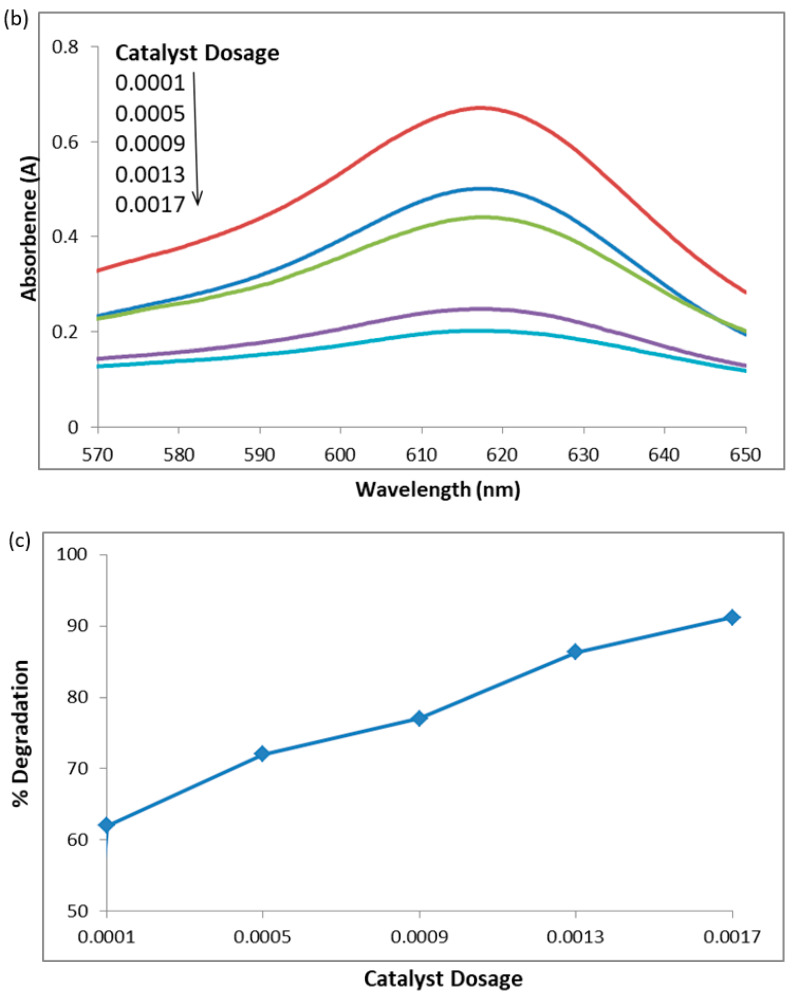
Photographic images of dilutions at different catalyst dosages (**a**) UV–visible graph of MG dye degraded with different amounts of photocatalyst for 60 min (**b**) and percentage degradation of malachite green dye for 60 min with different amounts of photocatalyst (**c**).

The sequence of photographic images visually captures the dilution phenomenon observed within solutions of the catalyst at varying dosages: 0.0001 g, 0.0005 g, 0.0009 g, 0.0013 g, and 0.0017 g, as shown in Figure 7a. These images provide a comprehensive view of how the dosage of the catalyst influences the appearance of the solutions. The effect of the catalyst dosage becomes more pronounced as the images progress. Solutions with higher catalyst dosages display a more noticeable change in color and intensity compared to those with lower dosages. This change in color is indicative of the catalyst’s influence on the degradation process. The impact of catalyst dosage on the photodegradation rate of the MG dye was investigated by varying the amount of catalyst ranging from 0.0001 g to a maximum of 0.0017 g per 20 mL of dye solution at a constant time of 60 min. In Figure 7b, the UV–visible graph depicts the degradation of MG in aqueous media containing different catalyst amounts. It is evident that the photodegradation rate of malachite green increases as the catalyst dosage increases. Initially, the degradation rate shows a rapid increase, followed by a gradual decrease, and eventually reaches a nearly constant level. This behavior may be attributed to the aggregation or agglomeration of catalyst particles, which reduces the availability of active surface sites. A higher catalyst dosage leads to an increased surface area that is available for catalyst interactions and a higher concentration of free radicals per milliliter of the MG solution. This, in turn, results in a higher percentage of dye removal (Bhushan et al., 2020). Figure 7c presents the percentage degradation graph of the MGn dye degraded using different amounts of the photocatalyst for 60 min. The percentage degradation values are 62%, 72%, 77%, 86%, and 91% when employing 0.0001 g, 0.0005 g, 0.0009 g, 0.0013 g, and 0.0017 g of the catalyst, respectively.

#### 2.2.5. Effect of Recovered Catalyst on Photodegradation

**Figure 8:**
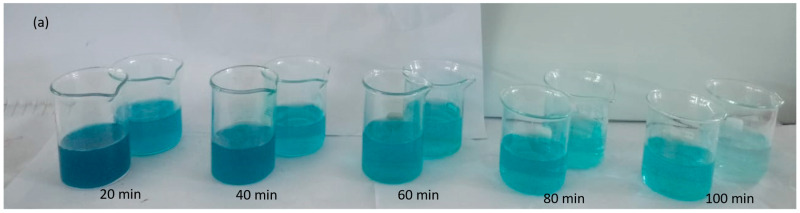

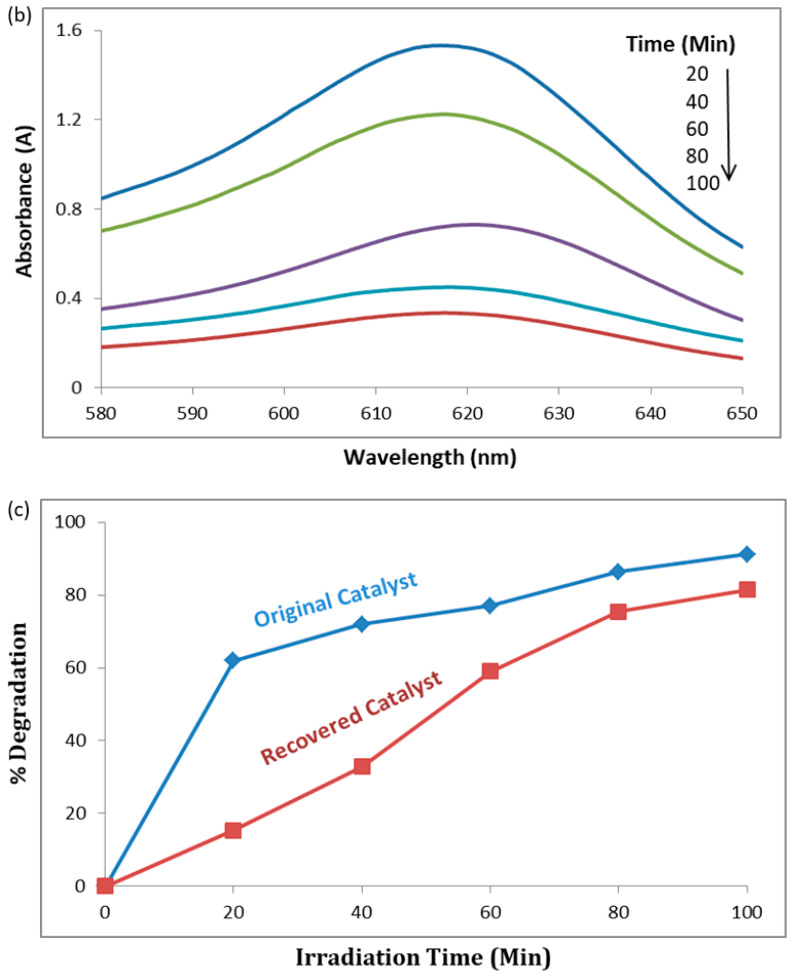
Malachite green dye degraded using the original catalyst (back row) and recovered catalyst (front row) at different time intervals (**a**). UV–visible graph of MG dye degradation with different doses of recovered photocatalyst (**b**), and percent degradation of MG dye by original and recovered photocatalyst (**c**).

The process of photocatalytically degrading MG dye was conducted using both the original catalyst (located in the back row) and the recovered catalyst (positioned in the front row), observed at various time intervals, as depicted in Figure 8a. After filtration, the recovered catalyst was thoroughly washed with distilled water to remove any adsorbed dye. Notably, distinct visual changes were observed in the appearance of the dye and its interaction with the catalysts. For the original catalyst, the dye’s vibrant color gradually faded, indicating its breakdown into simpler molecules owing to photocatalytic activity. As degradation continued, the dye’s color lightened. In contrast, the recovered catalyst led to a darker hue, suggesting less effective reactivity and reusability. The differing color outcomes highlight the distinct roles of catalysts in degradation, underlining their potential for efficient pollutant remediation. Figure 8b presents the UV–visible graph showing the photocatalytic activity of the recovered catalyst in degrading the MG dye, albeit with a lower efficiency compared to the original catalyst. The decrease in photocatalytic activity can be attributed to the active sites on the photocatalyst’s surface being blocked by the deposition of photoinsensitive hydroxides. Figure 8c displays the percentage of MG dye degradation by the original and recovered photocatalysts. The results indicate that the original catalyst degraded 91% of the dye, while the recovered catalyst achieved a degradation of 81% within a 60-min timeframe.

### 2.3. Proposed Mechanism for Photodegradation of MG Dye Degradation Using Ag-Mn Oxide Nanoparticles

**Figure 9:**
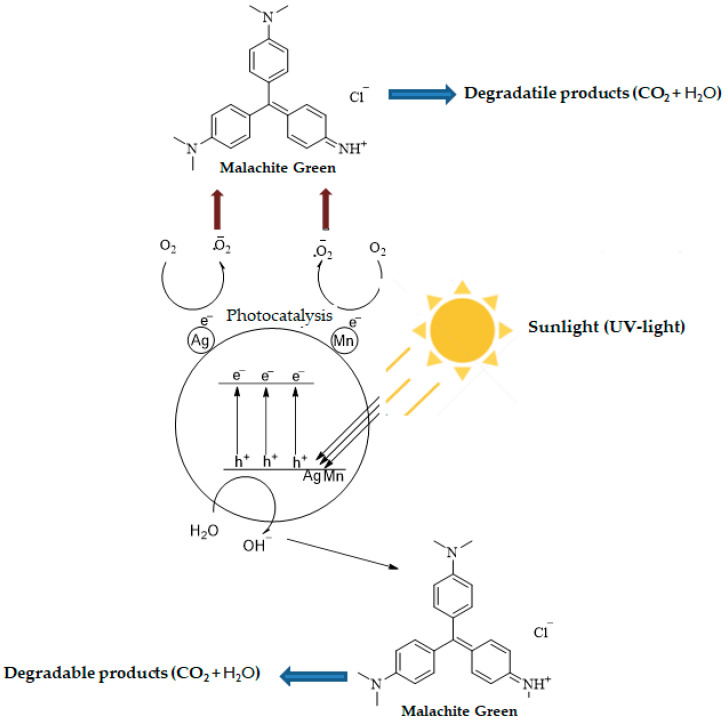
Proposed mechanism for the photocatalytic degradation of MG dye.

Photocatalytic degradation typically involves multiple stages, including adsorption–desorption, the generation of electron–hole pairs, recombination of these pairs, and chemical reactions [24]. However, the photocatalytic degradation of MG molecules comprises three primary steps: (1) efficient adsorption of MG molecules, (2) substantial absorption of incident light photons, and (3) initiation of charge-transfer reactions leading to the generation of oxidizing and reducing radicals [25]. Figure 9 shows the proposed mechanism for the photocatalytic degradation of the organic compound, malachite green dye. 

When sunlight irradiates the surface of photocatalysts (Ag-Mn-oxide nanoparticles), electrons (e^−^) in the valence band (VB) of the metals are excited to the conduction band (CB), resulting in the generation of positive holes (h^+^) in the valence band (VB). The positive holes either directly oxidize dye molecules to reactive intermediates owing to their high oxidative potential or react with water (H_2_O) molecules and produce hydroxyl radicals (**^∙^**OH). The electrons present in the conduction band of the metals react with O_2_ molecules and reduce them to superoxide anion radicals (**^∙^**O_2_**^−^**). These radicals are highly reactive and cause dye degradation. The important possible reactions are summarized in the following equations.

NPs + *hv* → NPs (h^+^ + e^−^) (1)

h^+^ + Dye → Oxidation of the dye molecules (2)

h^+^ + OH^−^/H_2_O → **^∙^**OH (3)

e^−^ + O_2_ → **^∙^**O_2_**^−^** (4)

**^∙^**OH + **^∙^**O_2_**^−^**+ dye → degradable/less-toxic species (5)

Various catalysts have been synthesized to enhance the efficiency of photocatalysis. Among these, metal-oxide nanoparticles stand out owing to their low toxicity and ease of transformation to hydroxides or oxides. An important attribute is the material’s bandgap, which determines the energy difference between the oxidation and reduction processes. The bandgap significantly influences the photocatalytic performance of nanomaterials, where a narrower bandgap corresponds to higher photocatalytic activity. An increase in light absorption accompanied by a redshift indicates that the photocatalyst maintains its performance over time, making it both reliable and cost-effective [26].

Photocatalysts are commonly produced using nanoparticles of metal oxides, such as TiO_2_, ZnO, Bi_2_O_3_, Fe_2_O_4_, WO_3_, CuO, Cu_2_O, and SnO_2_ [27]. These metal-oxide nanoparticles come in various shapes, including nanoparticles, nanospheres, nanofibers, nanotubes, nanoribbons, and nanosheets. In Table 1, a comparison is provided for the degradation of MG dye in previous studies using different metal-oxide nanoparticles, along with the experimental conditions employed.

### 2.4. Bandgap Energy Analysis

The addition of manganese to silver in the production of photocatalysts serves a crucial purpose driven by the intrinsic properties of these materials. Specifically, the energy difference between the valence band and the conduction band, known as the bandgap energy, plays a pivotal role in the catalytic activity of the resulting compound. Manganese possesses a bandgap energy of 1.3 eV [49], indicating its capacity to absorb light energy and promote electronic transitions. On the other hand, pure silver exhibits a significantly higher bandgap energy of 2.5 eV [50]. This higher energy requirement for electronic excitation in pure silver limits its effectiveness as a photocatalyst, resulting in reduced catalytic activity.

The strategic blending of manganese with silver nanoparticles addresses this limitation by lowering the overall bandgap energy of the composite material. This reduction in bandgap energy enables easier electronic excitation within the material upon light absorption. As a result, the photocatalytic degradation of the MG dye by the composite material is substantially enhanced. The introduction of manganese to the composite system also contributes to this enhancement. When manganese is present, the electrons within the silver nanoparticles can readily transition from the valence band to the conduction band of the manganese component. This transition occurs with minimal energy expenditure, owing to the favorable alignment of energy levels between manganese and silver.

In essence, the synergy between manganese and silver creates a photocatalyst with improved efficiency. By mitigating the energy barrier for electronic excitation through the reduction in the bandgap energy, the composite material becomes highly proficient at harnessing light energy to facilitate catalytic reactions. This phenomenon underscores the significance of using manganese in conjunction with silver for producing advanced photocatalysts that have superior performance characteristics.

An UV–Vis spectrophotometer was employed to investigate both the absorption region and optical activity of the prepared photocatalyst. To determine the optical bandgaps, a Tauc equation, represented by Equation (3), was utilized:

(*ahv*) _½_ = *A*(*hv* − *Eg*) (6)

**Figure 10:**
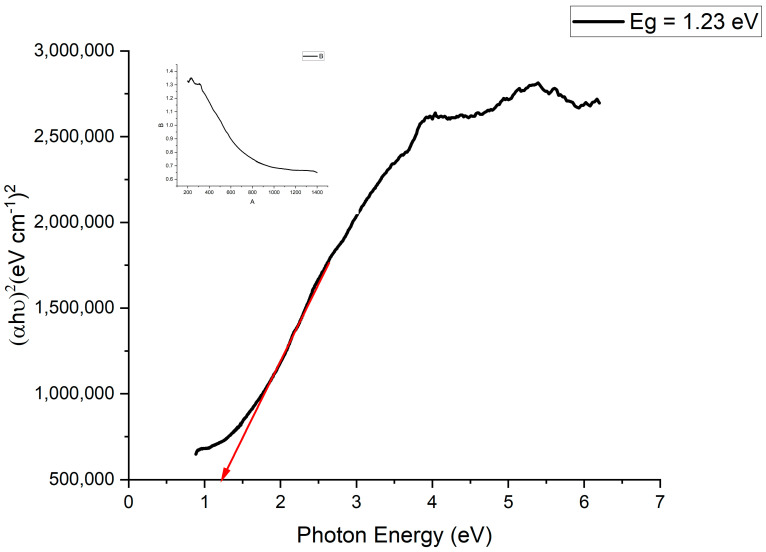
Bandgap plot of the prepared Ag-Mn oxide nanoparticles from the UV–vis DRS results.

By plotting (*αhv*) _1/2_ vs. *hv*, the extrapolation of a linear fit to the *X*-axis provides insights into the bandgaps (*Eg*). The application of a Tauc plot confirmed that the bandgap of the Ag-Mn nanoparticles was 1.23 eV, as visually depicted in Figure 10. This bandgap of 1.23 eV suggests that the prepared photocatalyst exhibits potential for visible-light utilization in photocatalytic processes [51].

## 3. Materials and Methods

### 3.1. Chemicals

Manganese chloride (MnCl_4_.2H_2_O), silver nitrate (AgNO_3_), sodium hydroxide (NaOH), nitric acid (HNO_3_), hydrochloric acid (HCl) and sulfuric acid (H_2_SO_4_) were purchased from Frontier Chemical Company, while malachite green dye was purchased from Danyal Trading Company at Mingora Pakistan.

### 3.2. Instrumentation

The morphological study of bimetallic oxide NPs was conducted using JEOL, JSM-5910 scanning electron microscopy (SEM). The elemental composition analysis of A-MWCNT-supported and unsupported bimetallic oxide NPs was performed using EDX (Model INCA 200/Oxford Instruments, Oxford, UK). The photodegradation study of malachite green in an aqueous medium was conducted using a UV–visible spectrophotometer. The X-ray diffraction (XRD) patterns of the prepared catalyst were collected using a PHILIPS X’ Pert PRO X-ray diffractometer and Cu K-α radiation. The range of XRD pattern collection was set from 10° to 80°, with a fine step increment of 0.01°.

### 3.3. Preparation of Silver–Manganese Oxide Nanoparticles

To prepare silver–manganese bimetallic oxide nanoparticles (Ag-Mn-NPs), a round-bottom flask was used to combine 100 mL of AgNO_3_ solution (0.1 M) and 100 mL of MnCl_4_.2H_2_O solution (0.1 M). Dropwise, a 0.2 M NaOH solution was added to the mixture while stirring continuously until a basic pH of 10 was reached. The resulting mixture was then heated to 60 °C and stirred for 2 h. During this process, the silver nitrate and manganese chloride underwent a chemical transformation and formed Ag-Mn oxide nanoparticles, which appeared as a precipitate. After cooling, the mixture was filtered, and the Ag-Mn oxide nanoparticles were thoroughly washed with distilled water multiple times to remove any remaining unreacted chemicals or impurities. Finally, the Ag-Mn oxide NPs were dried either in sunlight or using an oven and stored for further use.

### 3.4. Photodegradation of Malachite Green Dye Using Ag-Mn Oxide Nanoparticles

The Ag-Mn oxide nanoparticles prepared in this study were utilized as a photocatalyst for the degradation of malachite green (MG) dye under sunlight irradiation. A 25-ppm solution of malachite green dye was prepared, and 0.0001 g of Ag-Mn oxide nanoparticles was added separately to 20 mL of the dye solution in 50 mL beakers. The samples were allowed to reach adsorption–desorption equilibrium by keeping them in the dark for 20 min. Subsequently, the mixtures were exposed to sunlight and stirred continuously for different durations of irradiation time. After sunlight irradiation, the catalyst nanoparticles were separated from the dye solution using a filtration process. The degradation of MG dye was monitored using a UV–visible spectrophotometer. This study investigated the effects of the catalyst dosage, recovered catalyst duration, dye concentration, time, and medium pH on the photodegradation of MG dye.

In the catalyst dosage and recovered catalyst dosage studies, various amounts of the catalyst were used while keeping other parameters constant to examine their impacts on photodegradation. The pH study involved preparing different solutions at pH values of 4, 7, and 10 to assess the effect of pH on dye degradation. Acidic solutions were prepared by adding dropwise a 0.1 M HNO_3_ solution, while basic solutions were prepared by adding dropwise a 0.1 M NaOH solution to distilled water before preparing the dye solutions. The time study involved photodegrading the dye solution for different time intervals (20, 40, 60, 80, and 100 min) while maintaining other parameters constant. Similarly, in the dye concentration study, different solutions at concentrations of 15, 20, 25, 30, and 35 ppm were subjected to photodegradation under consistent conditions.

### 3.5. UV–Vis Analysis

The photodegradation study of the malachite green dye was conducted using a UV–vis spectrophotometer. The percentage degradation of dyes was calculated using the following formulas:

(7) $$\mathrm{Degradation}\mathrm{rate}\;(\%)=\left(\frac{C_{0}-C}{C_{0}}\right)\times 100$$

(8) $$\mathrm{Degradation}\mathrm{rate}\;(\%)=\left(\frac{A_{0}-A}{A_{0}}\right)\times 100$$

where *C*_0_ stands for initial dye concentrations, *C* stands for the concentration of the dye after sunlight irradiation, *A*_0_ shows the initial absorbance, and *A* is the dye absorbance after sunlight irradiation.

## 4. Conclusions

The present study found that Ag-Mn oxide nanoparticles exhibited strong photocatalytic activity for degrading malachite green dye. Within 100 min, approximately 92% of the dye was successfully degraded. Moreover, longer irradiation times led to increased dye degradation. Furthermore, the degradation of the MG dye was enhanced by employing larger amounts of the catalyst, as evidenced by the degradation of 91% of the dye using 0.0017 g of the photocatalyst over 60 min. Additionally, increasing the pH of the medium resulted in enhanced dye degradation, with 99% of the dye degraded in just 60 min at pH 10. Conversely, the photodegradation rate decreased with higher concentrations of the dye.

## Figures and Tables

**Table 1:** Some previous studies conducted for malachite green dye degradation by various metal-oxide-based nanoparticles.

Metal Oxide NM	Synthesis Technique	Morphology	Photocatalytic Experimental Setup	Degradation Efficiency [%]	Ref.
Xanthan gum/SiO_2_	Ultra-sonication with polymerization	Lobule	10 mg catalyst, 450 ppm of MG dye, pH = 7, the temperature of 30 °C, 480 min,	99.5	Abu Elella et al., 2021 [28]
CuMn_2_ O_4_	Co-precipitation method	Flake-like structure	Daylight, 60 min. UV light, 60 min, bandgap value 2.54 eV	94.80,	John Abel et al., 2019 [29]
Chitosan/TiO_2_	–	Spherical nanoparticles	70 ppm, 90 min, bandgap value ≈ 3 eV	90.70	Bahal et al., 2019 [30]
rGO/CuS	Co-precipitation method	Irregular hexagonal 100 mg photocatalyst, 10 ppm dye,		97.60	El-Hout et al., 2020 [31]
			under sunlight at room temperature, bandgap value 2 eV		
ZnFe_2_ O_4_	Probe sonication	Spongy like	Under sunlight, UV lamp, it took about 180 min, 2.4 eV	98–88	Surendra et al., 2020 [32]
Hematite	Combustion	Spherical and irregular structure	Presence of H_2_ O_2_, 20 ppm dye, UV source of 250 W, 0.1 g catalyst, 70 min, bandgap 1.45 eV	100	Alharbi and Abdelrahman, 2020 [33]
Cu_2_O	Sonochemical method	Uniform Icosahedron	10 ppm dye, 10 mg catalyst, visible lamp, 45 min, 2.26 eV	91.89	Muthukumaran et al., 2019 [34]
ZnO	Sol–gel method	Spherical structure	10 ppm dye concentration, 20 mg catalyst, UV lamp, 40 min, bandgap 3.3 eV	99	Sukri et al., 2020 [35]
ZnO	Green synthesis method	Irregular hexagon	100 ppm dye, 10 mm catalyst, 150 min, bandgap value 3.37 eV	Complete	Brindhadevi et al., 2020 [36]
BiOCl	Hydrolysis method	Tetragonal structure	Visible light, 120 min, pH 2.3 to 14, under normal room temperature, bandgap 3.2 eV	Remarkable	Sarwan et al., 2017 [37]
TiO_2_: Fe	Sol–gel method	Nanotubes, orthorhombic	Sunlight, 210 min, bandgap 2.57 eV	Complete	He, 201 [38])
CeO_2_	Chemical precipitation method	Cubic fluorite	Visible light, 80 min, bandgap value 2.90 eV	99	Kang et al., 2017 [39]
TiO_2_	Micro-emulsion method	Spherical	10 mg catalyst, 10 ppm dye, visible light, 50 min, bandgap 3.2 eV	96.40	Ma et al., 2018 [40]
Mn-doped ZnO	Wet diffusional impregnation	Tetragonal	3 g catalyst, 80 ppm concentration, visible light, 180 min, bandgap 3.2 eV	Faster degradation	Mohamed and Shawky, 2018 [41]
ZnO/CuO	Hydrothermal method	–	0.2 g catalyst, 15 ppm dye, pH-10, UV lamp, 240 min, bandgap value 4.42 eV	82	Batool, 2018 [42]
ZnO:Ag	Soft chemical method	Spherical structure	45 min, 500-Watt tungsten lamp, 60 min, visible light, pH 4.66, temperature of 30 °C, bandgap of 2.67 eV	88.8	Nithiyadevi and Ravichandran, 2017 [43]
SnIn_4_ S_8_	Solvothermal method	Spherical structure	500 kW Xe lamp, 30 min, at room temperature, bandgap 1.53 eV	Strong degradation	Lu et al., 2017 [44]
CuWO_4_-RGO	Hydrothermal method	Agglomerated with polycrystalline nature	2 ppm dye, 50 mg catalyst, 370 W mercury halide visible light, 60 min, 2.2 eV	93	Babu et al., 2020 [45]
CuWO_4_-GO	Ball-milling method	Microstructure	0.05 g catalyst, 10 ppm dye, visible lamp, 80 min,	95	Du et al., 2020 [46]
MnFe_2_ O_4_	Microwave-assisted combustion method	Irregular shape agglomerates	30 mg catalyst, 50 ppm dye, 60 min under natural pH condition	Maximum complete	de Andrade et al., 2021 [47]
Fe-Cu binary oxides	Electron spun method	Hair like structure	3 mg catalyst, 100 ppm dye solution, pH = 1, UV lamp, 60 min	91.40	Zafari et al., 2021 [48]
Ag-Mn- oxide nanoparticles	Wet chemical precipitation method		aqueous solution containing 25 ppm of MG, 100 min	92%	This study
		--	60 min of degradation, at pH 4, 7, and 10	34%, 72%, and 99% respectively	This study

## Data Availability

New data were produced and analyzed in this study. Data sharing is applicable to this article only on request.

## References

[1] Amdeha E., Makhlouf A.S.H., Ali G.A.M. (2021). Recovery of nanomaterials from agricultural and industrial wastes for water treatment applications. Waste Recycling Technologies for Nanomaterials Manufacturing.

[2] Mostafa E.M., Amdeha E. (2022). Enhanced photocatalytic degradation of malachite green dye by highly stable visible-light-responsive Fe-based tri-composite photocatalysts. Environ. Sci. Pollut. Res..

[3] Qiu R., Zhang D., Mo Y., Song L., Brewer E., Huang X., Xiong Y. (2008). Photocatalytic activity of polymer-modified ZnO under visible light irradiation. J. Hazard. Mater..

[4] Royer B., Cardoso N.F., Lima E.C., Macedo T.R., Airoldi C. (2010). A useful organofunctionalized layered silicate for textile dye removal. J. Hazard. Mater..

[5] Yadav R., Chundawat T.S., Rawat P., Rao G.K., Vaya D. (2021). Photocatalytic degradation of malachite green dye by ZnO and ZnO–β-cyclodextrin nanocomposite. Bull. Mater. Sci..

[6] Mahmoodi N.M., Salehi R., Arami M., Bahrami H. (2011). Dye removal from colored textile wastewater using chitosan in binary systems. Desalination.

[7] Kumar V.G., Gokavarapu S.D., Rajeswari A., Dhas T.S., Karthick V., Kapadia Z., Shrestha T., Barathy I.A., Roy A., Sinha S. (2011). Facile green synthesis of gold nanoparticles using leaf extract of antidiabetic potent Cassia auriculata. Colloids Surf. B Biointerfaces.

[8] Singh J., Dhaliwal A.S. (2020). Plasmon-induced Photocatalytic Degradation of Methylene Blue Dye Using Biosynthesized Silver Nanoparticles as Photocatalyst. Environ. Technol..

[9] Nagar N., Devra V. (2019). A Kinetic Study on the Degradation and Biodegradability of Silver Nanoparticles Catalyzed Methyl Orange and Textile Effluents. Heliyon.

[10] Kamat P. (2012). Manipulation of charge transfer across semiconductor interface a criterion that cannot be ignored in photo catalyst design. J. Phys. Chem. Lett..

[11] Hammad A., Haitham M., El-Bery H.M., EL-Shazly A.H., Elkady M. (2018). Effect of WO_3_ Morphological Structure on its Photoelectrochemical Properties. Int. J. Electrochem. Sci..

[12] Velusamy P., Lakshmi G. (2017). Enhanced Photocatalytic Performance of (ZnO/CeO_2_)-b-CD System for the Effective Decolorization of Rhodamine B under UV Light Irradiation. Appl. Water Sci..

[13] Saeed K., Khan I., Gul T., Sadiq M. (2017). Efficient Photodegradation of Methyl Violet Dye Using TiO_2_/Pt and TiO_2_/Pd Photocatalysts. Appl. Water Sci..

[14] Nasrollahzadeh M., Sajadi S.M., Maham M., Kohsari I. (2018). Biosynthesis, Characterization and Catalytic Activity of the Pd/Bentonite Nanocomposite for Base- and Ligand-Free Oxidative Hydroxylation of Phenylboronic Acid and Reduction of Chromium (VI) and Nitro Compounds. Microporous Microporous Mater..

[15] Zada N., Khan I., Shah T., Gul T., Khan N., Saeed K. (2020). Ag–Co oxides nanoparticles supported on carbon nanotubes as an effective catalyst for the photodegradation of Congo red dye in aqueous medium. Inorg. Nano-Metal Chem..

[16] Culp S.J., Beland F.A. (1996). Malachite green: A toxicological review. J. Am. Coll. Toxicol..

[17] Srivaji S., Sinha R., Roy D. (2004). Toxicological effects of malachite green. Aquat. Toxicol..

[18] Sarvamangala D., Kondala K., Sivakumar N., Saratchandra Babu M., Manga S. (2013). Synthesis, characterization and anti microbial studies of AgNP’s using pro-biotics. Int. Res. J. Pharm..

[19] Jaast S., Grewal A. (2021). Green synthesis of silver nanoparticles, characterization and evaluation of their photocatalytic dye degradation activity. Curr. Res. Green Sustain. Chem..

[20] Wang W., Yu J., Xiang Q., Cheng B. (2016). Enhanced photocatalytic activity of hierarchical macro/mesoporous TiO_2_–graphene composites for photodegradation of acetone in air. Appl. Catal. B Environ..

[21] Hachem C., Bocquillon F., Zahraa O., Bouchy M. (2001). Decolourization of textile industry wastewater by the photocatalytic degradation process. Dyes Pigments.

[22] Sohrabi M., Davallo M., Miri M. (2009). Influence of operational parameters on eliminating azo dyes from wastewater by advanced oxidation technology. Int. J. Chem. Tech. Res..

[23] Fu H., Yang Y., Zhu R., Liu J., Usman M., Chen Q., He H. (2018). Superior adsorption of phosphate by ferrihydrite-coated and lanthanum-decorated magnetite. J. Colloid Interface Sci..

[24] Elkady M.F., Hassan H.S. (2021). Photocatalytic degradation of malachite green dye from aqueous solution using environmentally compatible Ag/ZnO polymeric nanofibers. Polymers.

[25] Abukhadra M.R., Shaban M., Abd El Samad M.A. (2018). Enhanced photocatalytic removal of Safranin-T dye under sunlight within minute time intervals using heulandite/polyaniline@ nickel oxide composite as a novel photocatalyst. Ecotoxicol. Environ. Saf..

[26] Sundar K.P., Kanmani S. (2020). Progression of Photocatalytic reactors and it’s comparison: A Review. Chem. Eng. Res. Des..

[27] Krishna Kumar A.S., Warchol J., Matusik J., Tseng W.L., Rajesh N., Bajda T. (2022). Heavy metal and organic dye removal via a hybrid porous hexagonal boron nitride-based magnetic aerogel. NPJ Clean Water.

[28] Abu Elella M.H., Goda E.S., Gamal H., El-Bahy S.M., Nour M.A., Yoon K.R. (2021). Green antimicrobial adsorbent containing grafted xanthan gum/SiO_2_ nanocomposites for malachite green dye. Int. J. Biol. Macromol..

[29] John Abel M., Pramothkumar A., Senthilkumar N., Jothivenkatachalam K., Fermi Hilbert Inbaraj P., Joseph prince J. (2019). Flake-like CuMn_2_O_4_ nanoparticles synthesized via co-precipitation method for photocatalytic activity. Phys. B Condens. Matter.

[30] Bahal M., Kaur N., Sharotri N., Sud D. (2019). Investigations on amphoteric chitosan/TiO_2_ bionanocomposites for application in visible light induced photocatalytic degradation. Adv. Polym. Technol..

[31] El-Hout S.I., El-Sheikh S.M., Gaber A., Shawky A., Ahmed A.I. (2020). Highly efficient sunlight-driven photocatalytic degradation of malachite green dye over reduced graphene oxide-supported CuS nanoparticles. J. Alloys Compd..

[32] Surendra B.S., Shashi Shekhar T.R., Veerabhadraswamy M., Nagaswarupa H.P., Prashantha S.C., Geethanjali G.C., Likitha C. (2020). Probe sonication synthesis of ZnFe_2_O_4_ NPs for the photocatalytic degradation of dyes and effect of treated wastewater on growth of plants. Chem. Phys. Lett..

[33] Alharbi A., Abdelrahman E.A. (2020). Efficient photocatalytic degradation of malachite green dye using facilely synthesized hematite nanoparticles from Egyptian insecticide cans. Spectrochim. Acta-Part A Mol. Biomol. Spectrosc..

[34] Muthukumaran M., Gnanamoorthy G., Varun Prasath P., Abinaya M., Dhinagaran G., Sagadevan S., Mohammad F., Oh W.C., Venkatachalam K. (2019). Enhanced photocatalytic activity of Cuprous Oxide nanoparticles for malachite green degradation under the visible light radiation. Mater. Res. Express.

[35] Sukri S.N.A.M., Isa E.D.M., Shameli K. (2020). Photocatalytic Degradation of Malachite Green Dye by Plant-mediated Biosynthesized Zinc Oxide Nanoparticles. IOP Conference Series: Materials Science and Engineering.

[36] Brindhadevi K., Samuel M.S., Verma T.N., Vasantharaj S., Sathiyavimal S., Saravanan M., Pugazhendhi A., Duc P.A. (2020). Zinc oxide nanoparticles (ZnONPs)induced antioxidants and photocatalytic degradation activity from hybrid grape pulp extract (HGPE). Biocatal. Agric. Biotechnol..

[37] Sarwan B., Acharya A.D., Pare B. (2017). Visible light-driven photocatalytic degradation and mineralization of the malachite green dye in a slurry photoreactor. Part. Sci. Technol..

[38] He H.Y. (2017). Facile synthesis of Bi_2_S_3_ nanocrystalline-modified TiO_2_: Fe nanotubes hybrids and their photocatalytic activities in dye degradation. Part. Sci. Technol..

[39] Kang L., Zhang Y.J., Zhang K., Zhang L., Yang M.Y. (2017). Photocatalytic degradation of malachite green by a novel CeO_2_ loaded alkali-activated steel slag-based nanocomposite. Integr. Ferroelectr..

[40] Ma Y., Ni M., Li S. (2018). Optimization of malachite green removal from water by TiO_2_ nanoparticles under UV irradiation. Nanomaterials.

[41] Mohamed R.M., Shawky A. (2018). CNT supported Mn-doped ZnO nanoparticles: Simple synthesis and improved photocatalytic activity for degradation of malachite green dye under visible light. Appl. Nanosci..

[42] Batool M. (2018). Biosynthesis of Copper Nanoparticles by using Aloe Barbadensis Leaf Extracts. Interv. Pediatr. Dent. Open Access J..

[43] Nithiyadevi K., Ravichandran K. (2017). Enhancement of photocatalytic and antibacterial activities of ZnO:Ag nanopowders through the addition of bamboo charcoal: An efficient natural adsorbent. Acta Metall. Sin. (Engl. Lett.).

[44] Lu M., Wang X., Zhang Y., Li Z., Xu S., Yao C. (2017). Facile synthesis of a symmetrical squarylium dye sensitized SnIn4S8 composites with enhanced photocatalytic activity under visible-light irradiation. J. Mater. Sci. Mater. Electron..

[45] Babu M.J., Botsa S.M., Rani S.J., Venkateswararao B., Muralikrishna R. (2020). Enhanced photocatalytic degradation of cationic dyes under visible light irradiation by CuWO_4_-RGO nanocomposite. Adv. Compos. Hybrid Mater..

[46] Du F., Sun L., Huang Z., Chen Z., Xu Z., Ruan G., Zhao C. (2020). Electrospun reduced graphene oxide/TiO_2_/poly(acrylonitrile-co-maleic acid) composite nanofibers for efficient adsorption and photocatalytic removal of malachite green and leucomalachite green. Chemosphere.

[47] Andrade F.V., de Oliveira A.B., Siqueira G.O., Lage M.M., de Freitas M.R., de Lima G.M., Nuncira J. (2021). MnFe_2_O_4_ nanoparticulate obtained by microwave-assisted combustion: An efficient magnetic catalyst for degradation of malachite green cationic dye in aqueous medium. J. Environ. Chem. Eng..

[48] Zafari S.H., Saadatjou N., Shaabani B. (2021). Fe-Cu binary oxides as low-cost adsorbents and their application to photocatalytic removal of Acid Red 14, Methyl Orange, and Malachite Green from aqueous solutions. J. Appl. Chem..

[49] Zaanen J., Sawatzky G.A., Allen J.W. (1985). Band gaps and electronic structure of transition-metal compounds. Phys. Rev. Lett..

[50] Aziz A., Khalid M., Akhtar M.S., Nadeem M., Gilani Z.A., Ul Huda Khan Asghar H.M.N., Rehman J., Ullah Z., Saleem M. (2018). Structural, morphological and optical investigations of silver nanoparticles synthesized by sol-gel auto-combustion method. Dig. J. Nanomater. Biostruct..

[51] Sharfalddin A., Alzahrani E., Alamoudi M. (2016). Micro, Sono, Photocatalytic Degradation of Eosin B Using Ferric Oxide Doped with Cobalt. Am. Chem. Sci. J..

